# An assessment of dialysis provider’s attitudes towards timing of dialysis initiation in Canada

**DOI:** 10.1186/2054-3581-1-3

**Published:** 2014-04-07

**Authors:** Bikaramjit S Mann, Braden J Manns, Allison Dart, Joanne Kappel, Anita Molzahn, David Naimark, Sharon J Nessim, Steven Soroka, Michael Zappitelli, Manish M Sood, on behalf of the Canadian Kidney Knowledge Translation and Generation Network (CANN-NET)

**Affiliations:** Department of Medicine, University of Calgary, Calgary, Alberta Canada; Manitoba Institute of Child Health, University of Manitoba, Winnipeg, Manitoba Canada; Saskatoon Health Region, University of Saskatchewan, Saskatoon, Saskatchewan Canada; Faculty of Nursing, University of Alberta, Edmonton, Alberta Canada; Division of Nephrology, Sunnybrook and Women’s College Health Sciences Centre, Toronto, Ontario Canada; 7Jewish General Hospital, McGill University, Montreal, Quebec Canada; Department of Medicine, Dalhousie University, Halifax, Nova Scotia Canada; McGill University Health Centre, Montreal Children’s Hospital, Montreal, Quebec Canada; The Ottawa Hospital Research Institute, University of Ottawa, Ottawa, Ontario Canada

## Abstract

**Background:**

Physicians’ perceptions and opinions may influence when to initiate dialysis.

**Objective:**

To examine providers’ perspectives and opinions regarding the timing of dialysis initiation.

**Design:**

Online survey.

**Setting:**

Community and academic dialysis practices in Canada.

**Participants:**

A nationally-representative sample of dialysis providers.

**Measurements and Methods:**

Dialysis providers opinions assessing reasons to initiate dialysis at low or high eGFR. Responses were obtained using a 9-point Likert scale. Early dialysis was defined as initiation of dialysis in an individual with an eGFR greater than or equal to 10.5 ml/min/m^2^. A detailed survey was emailed to all members of the Canadian Society of Nephrology (CSN) in February 2013. The survey was designed and pre-tested to evaluate duration and ease of administration.

**Results:**

One hundred and forty one (25% response rate) physicians participated in the survey. The majority were from urban, academic centres and practiced in regionally administered renal programs. Very few respondents had a formal policy regarding the timing of dialysis initiation or formally reviewed new dialysis starts (N = 4, 3.1%). The majority of respondents were either neutral or disagreed that late compared to early dialysis initiation improved outcomes (85-88%), had a negative impact on quality of life (89%), worsened AVF or PD use (84-90%), led to sicker patients (83%) or was cost effective (61%). Fifty-seven percent of respondents felt uremic symptoms occurred earlier in patients with advancing age or co-morbid illness. Half (51.8%) of the respondents felt there was an absolute eGFR at which they would initiate dialysis in an asymptomatic patient. The majority of respondents would initiate dialysis for classic indications for dialysis, such as volume overload (90.1%) and cachexia (83.7%) however a significant number chose other factors that may lead them to early dialysis initiation including avoiding an emergency (28.4%), patient preference (21.3%) and non-compliance (8.5%).

**Limitations:**

25% response rate.

**Conclusions:**

Although the majority of nephrologists in Canada who responded followed evidence-based practice regarding the timing of dialysis initiation, knowledge gaps and areas of clinical uncertainty exist. The implementation and evaluation of formal policies and knowledge translation activities may limit potentially unnecessary early dialysis initiation.

**Electronic supplementary material:**

The online version of this article (doi:10.1186/2054-3581-1-3) contains supplementary material, which is available to authorized users.

## Résumé

**Contexte:**

Les perceptions et opinions des médecins peuvent influencer le moment d’initiation de la dialyse.

**Objectif:**

Examiner les perspectives et opinions des médecins relatives au moment d’initiation de la dialyse.

**Type d’étude:**

Sondage en ligne.

**Échantillon:**

Unités de dialyse communautaires et académiques au Canada.

**Participants:**

Un échantillon représentatif de médecins au Canada.

**Méthodes et instruments de mesure:**

Nous avons recueilli les opinions des professionnels impliqués en dialyse sur l'initiation de la dialyse basée sur le niveau de eDGF (inférieur ou supérieur à 10,5 mL/min/m^2^) grâce à un sondage envoyé à tous les membres de la Société canadienne de néphrologie en février 2013. Les réponses aux questions étaient exprimées par une échelle de Likert à 9 catégories. Nous avons préalablement testé le sondage afin d'évaluer sa durée et sa facilité d'administration. L'initiation précoce de la dialyse était définie par un début de dialyse en présence d'un eDGF supérieur ou égal à 10,5 mL/min/m^2^.

**Résultats:**

Cent quarante et un (taux de réponse de 25%) médecins ont participé au sondage. La majorité provenait de centres urbains et académiques et pratiquait dans des programmes régionaux de suppléance rénale. Très peu de répondants avaient un protocole formel pour le début de la dialyse ou avaient révisés les nouvelles initiations de dialyse (n = 4, 3,1%). La majorité des répondants était soit neutre ou en désaccord avec l’affirmation que l’initiation tardive, comparée à l’initiation précoce, améliore les issues (85-88%), réduit l’utilisation d’une FAV ou de la dialyse péritonéale (84-90%), conduit vers des patients plus malades (83%), ou était rentable (61%). Cinquante-sept pour cent des répondants estimaient que les symptômes urémiques apparaissent plus tôt chez les patients âgés ou souffrant de comorbidités. La moitié (51,8%) des répondants estimait qu’il existe un seuil de DFG où ils débuteraient la dialyse chez un patient asymptomatique. La majorité des répondants initierait la dialyse pour les indications classiques de dialyse, telles que la surcharge volémique (90,1%) et la cachexie (83,7%). Cependant, un nombre significatif de répondants ont rapporté d’autres facteurs qui les conduiraient à initier la dialyse précocement, incluant éviter une urgence (28,4%), la préférence du patient (21,3%) et l’inobservance (8,5%).

**Conclusions:**

Bien que la majorité des néphrologues au Canada ait répondu selon les lignes directrices basées sur les données probantes pour le moment d’initiation de la dialyse, des lacunes de connaissance et des incertitudes cliniques existent. La mise en œuvre et l’évaluation de politiques formelles et d’activités de valorisation des connaissances pourraient limiter l’initiation de dialyse précoce non nécessaire.

## Introduction

There is uncertainty regarding the optimal timing of the elective initiation of dialysis among patients being followed in clinic for progressive chronic kidney disease (CKD). Contrary to a previous opinion-based guideline recommendation that dialysis should be started earlier in the course of CKD [1], the findings of several observational studies suggest no discernable benefit or even a potential increase in mortality among those started on dialysis with a higher estimated glomerular filtration rate (eGFR) [2–5]. Furthermore, the IDEAL study, a randomized controlled trial, demonstrated that dialysis initiation at a higher eGFR (10 to 14 mL/min/1.73 m^2^) was not associated with a survival benefit when compared to initiating dialysis at a lower eGFR (5 to 7 mL/min/1.73 m^2^) in patients with end-stage renal disease (ESRD). The effect of early dialysis initiation among subgroups with co-morbid illnesses such as diabetes and heart disease was similar to the overall result.

Despite the lack of evidence supporting the initiation of dialysis earlier in the course of progressive CKD, that is at a higher eGFR value, the fraction of incident dialysis patients starting dialysis with an eGFR > 10 mL/min/1.73 m^2^ increased from 15% in 1996 to 30% in 2005 in the United States and 28% to 36% between 2001 and 2007 in Canada [6, 7]. More recent secular trends suggest the early observational studies and the results from the IDEAL trial in 2010 may be reversing this trend [8]. It remains unclear whether the improved Canadian and American trends are truly evidence-based or due to a host of other factors such as differences in regional practice, patient preference, changing patient comorbidities, limitations with the IDEAL study and it’s applicability to individual patients or the attitudes of nephrologists throughout Canada [9, 10].

The goal of this study was to survey Canadian nephrologists to assess their attitudes regarding the evidence surrounding timing of dialysis initiation and regarding the perceived advantages and disadvantages of initiating dialysis with a low eGFR *versus* a high eGFR.

## Methods

### Survey design

We developed a detailed survey to evaluate the demographics, practice patterns and opinions of nephrologists regarding the timing of dialysis initiation in advanced CKD patients (Additional file 1). BJM, DN, & MMS were principally involved with the survey questions and design. Pre-testing to evaluate approximate duration and ease of administration was completed by the survey designers and other study investigators (SS, DN). The final version of the survey was administered using a web-based survey program (SurveyMonkey™; Palo Alto, CA).

### Study population

All members of the Canadian Society of Nephrology (CSN) were contacted via email and asked to participate by means of an online survey in early 2013. One reminder email was sent to all CSN members and consent was assumed based on participation. Ethics approval was obtained by the St. Boniface Hospital regional ethics board and the University of Manitoba Bannatyne campus Research Ethics Board.

### Data collection and definitions

We defined early start dialysis as initiating dialysis with an eGFR ≥10.5 mL/min/1.73 m^2^ and late dialysis was defined as an eGFR <10.5 mL/min/1.73 m^2^. These cut-offs were based on data from the CANUSA study which helped to establish a recommended PD target for weekly Kt/V urea of 2, translating roughly to an eGFR of 10.5 mL/min/1.73 m^2^[11]. Participant demographic information, and practice characteristics were collected. Survey questions assessed reasons participants initiated dialysis at low eGFR or higher eGFR values. Responses to questions were obtained by using a 9-point Likert scale.

### Statistical analyses

Descriptive statistics were used for the data analysis. Differences between groups were evaluated using chi-square or Fisher’s exact tests as appropriate. Likert scale responses were categorized as 1–3 (disagree), 4–6 (neutral) and 7–9 (agree). Analysis was performed using STATA Version 13® (StataCorp LB, College Station, TX). A *p*-value of <0.05 was considered to be statistically significant.

## Results

The survey was sent to 564 Canadian nephrologists of whom141 (25%) responded. Table 1 outlines the demographics and practice characteristics of the participants. Thirty-two percent of the participants practice in Ontario, 22% in Quebec, 18% in Alberta, and 13% in British Columbia. Most nephrologists practice in urban (92%) and academic centres (67%). Length of time in practice was uniformly distributed and a majority were involved in continuing medical educational activities such as attending conferences and journal reading.

**Table 1:** **Demographics and practice characteristics of Canadian nephrologists who participated in the survey**

Characteristic	Respondents (n)	Percentage
**Provinces**		
British Columbia	18	12.9
Alberta	26	18.4
Saskatchewan	5	3.5
Manitoba	8	5.7
Ontario	46	32.6
Quebec	31	22.0
Nova Scotia	4	2.8
New Brunswick	2	1.4
Newfoundland	1	0.7
**Years in practice**		
0 – 5	20	14.1
6 – 10	38	27
11 – 15	32	22.7
16 – 20	18	12.8
>20	33	23.4
**Practice environment**		
Academic centre	95	67.4
Community	34	24.1
Mixture of both	12	8.5
**Estimated size of population served**		
<50,000	1	0.7
50,000 – 200,000	21	15.0
200,000 – 500,000	34	24.3
>500,000	84	67.1
**Estimated distribution of dialysis modality (population treated with each modality per centre)**		
Peritoneal Dialysis (PD)^§^		
0 – 100	78	63.9
101 – 200	39	32.0
>200	5	4.1
Hemodialysis (HD)^¶^		
0 – 250	57	46.3
251 – 500	37	30.1
>500	29	23.6
Stage 5 CKD Not on Dialysis*		
0 – 50	35	31.3
51 – 100	15	13.4
101 – 150	6	5.4
151 – 200	10	8.9
201 – 250	3	2.7
>250	43	38.4
**Use of smartphone or PDA in practice**		
Yes	103	73.0
No	38	27.0
**Use of electronic medical record in practice**		
Yes	99	70.2
No	42	29.8
**Journal reading (hours per week)**		
0	1	0.7
1-2	75	53.2
3-4	39	27.7
>4	26	18.4
**Continuing medical education (hours per month)** ^**✜**^		
0	2	1.4
1-2	19	13.6
3-4	37	26.4
>4	82	58.6
**Conferences (per year)**		
0	1	0.7
1	34	24.1
2	56	39.7
3	24	17.0
>3	26	18.4

Note: Total number of survey respondents n = 141. Unless otherwise stated confidence intervals were calculated using the total number of survey respondents ^§^122 respondents. ^¶^123 respondents. *112 respondents ^✜^140 respondents. HD, hemodialysis. This includes home nocturnal, in-centre short hemodialysis, and long conventional hemodialysis (described as >5 hours three times weekly), PDA, personal digital assistant.

Eighty-four percent of participants estimated that their hospitals or institutions provided care for a regional population of greater than or equal to 200,000 individuals.

**Figure 1:**
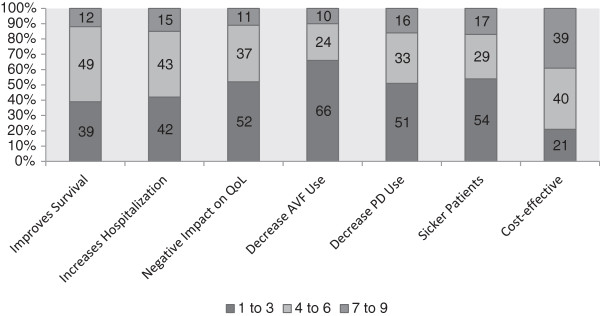
**Nephrologists’ opinions about initiating dialysis at a lower (<10.5 ml/min) compared to a higher (≥10.5 ml/min) eGFR.** Note: Number in bars represents percentages, 7–9 agree, 4–6 neutral, and 1–3 disagree.

**Figure 2:**
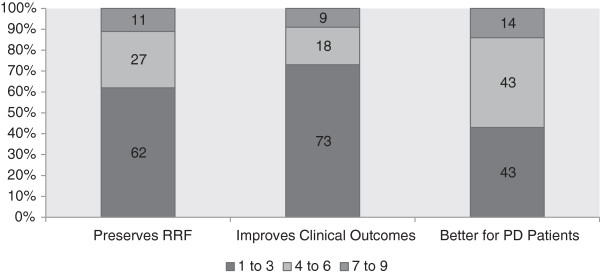
**Nephrologists’ opinions regarding initiation of dialysis at high (≥10.5 ml/min) compared to a low eGFR (<10.5 ml/min).** Note: Number in bars represents percentages PD, peritoneal dialysis; QoL, quality of life; AVF, arteriovenous fistula; RRF, residual renal function, 1–3 disagree, 4–6 neutral, 7–9 agree.

The majority (79%) of participants practice in a regionally administered renal program with a group education program for patients regarding dialysis, a pre-emptive transplant program, a dialysis modality coordinator and a multi-disciplinary vascular access clinic (Table 2). Very few participants (n = 4, 3.1%) reported a formal policy in their renal program with respect to timing of dialysis initiation (Table 2). Fifty-one percent (n = 64) of participants indicated they would initiate dialysis at a higher eGFR in patients with multiple comorbidities and 57% (n = 72) agreed that uremic symptoms occur earlier in patients with advanced age or a greater number of co-morbid conditions. The majority of participants were neutral or disagreed that initiation of dialysis in patients at a lower eGFR compared to a higher eGFR improves outcomes, worsens quality of life, decreases AVF or PD use and leads to sicker patients (see Figure 1). When asked whether initiation at higher eGFR compared to lower eGFR preserves residual renal function, improves clinical outcomes or is better for peritoneal dialysis patients 63, 73 and 43%, respectively disagreed (Figure 2). One-fifth disagreed that starting dialysis at an eGFR < 10.5 ml/min was cost effective. When asked about the results from the IDEAL trial, 48% agreed that results made them more likely to delay dialysis initiation while 18% disagreed.

**Table 2:** **Characteristics of renal program and policies for nephrologists in Canada who participated in the survey**

Characteristic*	N	%
Regionally administered renal program	107	76.6
Group education program for patients starting dialysis	129	95.6
Multidisciplinary vascular clinic	115	85.2
Pre-emptive transplant program	119	88.2
Modality coordinator	94	69.6
Rounds where dialysis modality selection discussed	44	32.8
Physician reimbursement equal for all dialysis modalities	66	49.3
Higher remuneration fee for managing patients with severe CKD	9	6.7
Policy for timing dialysis in place at renal program	4	3.1

Note: *The number of respondents for each question varied from 129 to 135; N number of participants responding Yes, % percentage of total respondents for that question.

**Figure 3:**
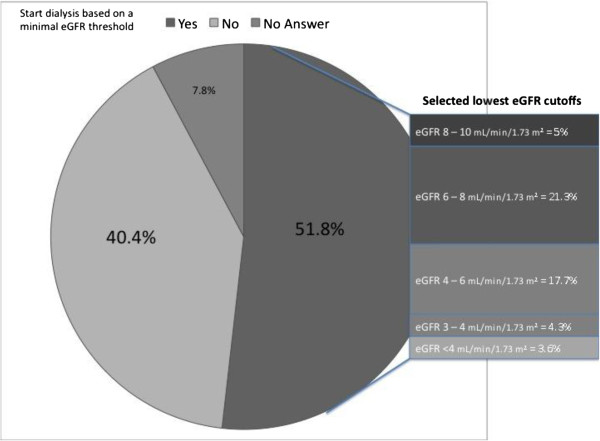
**Absolute lowest eGFR that Canadian nephrologists would initiate dialysis in asymptomatic patients.**

**Figure 4:**
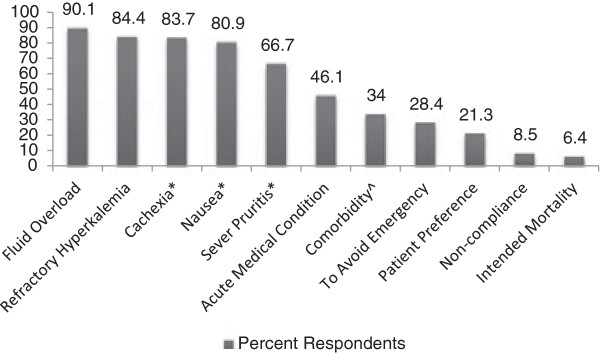
**Factors that participants indicated would increase the likelihood of deciding to initiate dialysis in patients with a high eGFR (≥10.5 ml/min).** Note: *Not explained by other etiologies, ^Comorbidities: Heart failure/coronary artery disease/diabetes/cirrhosis/age, Numbers above bars represents percentages

A minority of respondents (40.4%) indicated that there was no absolute lowest eGFR value which would mandate dialysis initiation in asymptomatic patients, while 51.8% of those who responded felt that there was such a limit. The most common eGFR value chosen as a limit was between 4 and 8 mL/min/1.73 m^2^ (Figure 3). The most common clinical factors that respondents indicated would prompt consideration of initiating dialysis at higher eGFR included classic indications such as fluid overload (90.1%), hyperkalemia refractory to medical therapy (84.4%) and uremic symptoms such as cachexia (83.7%), nausea (80.9%), and severe, otherwise unexplained, pruritis (66.7%, see Figure 4). Non-classical indications contributed a smaller proportion of the responses including avoidance of an emergent dialysis start (28.4%), patient preference (21.3%), and non-compliance (8.5%).

## Discussion

In this national survey study of Canadian nephrologists, we identified areas of clinical uncertainty that may be amendable to modification by appropriate knowledge translation activities. Of Canadian nephrologists surveyed, very few practiced in centres with a formal policy regarding dialysis initiation or a process to review all new dialysis starts. Fifty percent of participants have an absolute lowest eGFR at which they would start dialysis, and believe age and co-morbidity lead to earlier uremic symptoms. The majority disagreed that late dialysis improved clinical outcomes or worsened AVF or PD use but one-fifth disagreed it was cost-effective. Almost half agreed that results from the IDEAL trial would encourage them to delay dialysis initiation. These results suggest significant clinical uncertainty in regards to the timing of dialysis initiation among Canadian nephrologists. The implementation of formal policies and review systems for new dialysis starts may decrease this uncertainty and possibly reduce early dialysis initiation.

A recent European survey, sent to nephrologists as well as non-nephrologists, assessed the decision to initiate dialysis and found that 54% of respondents felt that, for uncomplicated patients, excretory kidney function was the most important factor to consider in the decision to initiate dialysis, with a median eGFR threshold of 10 mL/min/1.73 m^2^[12]. The survey also demonstrated that dialysis was initiated at higher eGFR values in private and for-profit centres. In contrast, the current survey was only sent to nephrologists and found that 51.4% of respondents felt that among asymptomatic patients, an eGFR between 4 and 8 mL/min/1.73 m^2^ was the level of excretory function at which they would initiate dialysis. The European survey also found that 86% of respondents believed that starting dialysis at an eGFR >10.5 mL/min/1.73 m^2^ was only beneficial in the presence of clinical signs and symptoms. Our survey results highlighted that respondents did not feel that initiating dialysis at low eGFR values (< 10.5 mL/min/1.73 m^2^) improved clinical outcomes, or preserved residual renal function.

Among respondents of the current survey, the results of the IDEAL trial appear to have less of an impact than what one might expect given that it is the only randomized trial addressing the issue of the appropriate eGFR to trigger the start of dialysis. However, nephrologists did feel that uremic symptoms occur earlier in those with comorbidities and it is possible that patients are exhibiting uremic symptoms at higher eGFR values in the real world, which would be consistent with the European survey [12], and may in part explain the lack of impact of the results from the IDEAL trial in clinical practice. It should also be pointed out that patients in the IDEAL trial initiated dialysis at higher levels of eGFR compared with their study protocol due to the development of uremic symptoms at higher eGFR values – 9.8 mL/min/1.73 m^2^ in the trial vs. 5 to 7 mL/min/1.73 m^2^ in the protocol [13]. This finding from the IDEAL trial highlights the difficulty in discerning symptoms of uremia from those related to other chronic diseases [13, 14]. At present there remains a limited amount of evidence regarding the signs and symptoms of early uremia and their association with patients-related outcomes. Furthermore an economic evaluation of the IDEAL trial identified an increase in cost of roughly CAN $18,000 for early start dialysis compared to late, a finding that roughly one-fifth of respondents were unaware of or in disagreement with [15]. An important point is that in the IDEAL trial, the eGFR in the primary analysis was calculated using the Cockcroft-Gault equation that is known to have low accuracy in late stage CKD unless corrected for bias [16]. Although the study authors performed a sensitivity analysis using the MDRD equation, the primary results as reported in the study abstract are often emphasized and may lead to confusion. In our survey, we did not clarify a preferred equation for estimating GFR.

The spectrum of responses among the Canadian nephrology community suggests significant clinical uncertainty regarding the optimal time to initiate chronic dialysis. This uncertainty is in keeping with other jurisdictions (US, Europe) where over the last decade patients are consistently being initiated with higher levels of eGFR [1, 4, 11]. The lack of clarity due to the high degree of patient cross over among the treatment arms of the IDEAL trial further adds to this uncertainty. As there are no imminent plans for another RCT and there is a lack of an objective uremia assessment tool, we feel nephrologists should apply the best evidence to date, namely that there is no demonstrable benefit (and potential harm) from early dialysis initiation, even among patients with comorbidity and it is not cost effective.

The synthesis and dissemination of the best evidence from research to front line clinicians is challenging [17]. One of the methods to overcome this barrier may be to utilize effective knowledge translation strategies [18, 19]. The CAnadian KidNey KNowledge TraNslation and GEneration NeTwork (CANN-NET), a pan Canadian collaboration to improve knowledge translation in Nephology, was able to identify an important clinical question, namely the timing of initiation of dialysis in CKD as the first step in their knowledge translation strategy. In the knowledge-to-action cycle our survey’s role was to identify barriers to knowledge use, such as the results of the IDEAL trial. To synthesize the state of current knowledge, a systematic review and recently guidelines were developed regarding the initiation of dialysis in patients with progressive CKD [20]. Utilizing the CANN-NET framework, a series of knowledge translation strategies will be implemented to disseminate the Canadian guidelines in an effort to optimize practice. Possible dissemination strategies include the use of novel technologies and facility-level practice changes. For example a smartphone application [21] that provides a point-of-care summary of these clinical practice guidelines may be useful as 73% of nephrologists report using a smartphone or PDA device. Our study also found that few centres have a process by which they determine the timing of dialysis initiation. Facility-level practice changes such as multidisciplinary rounds may provide a framework for a formal review prior to initiation of dialysis to help ensure that clinical and non-clinical factors have been fully considered prior to initiating dialysis. Lastly, many healthcare systems have attempted to alter practice patterns to improve evidence-based medicine and cost-effectiveness by altering physician remuneration. For example by financially compensating nephrologists for managing complex ESRD patients who are not on dialysis may, in turn, reduce early dialysis initiation. Only 6.7% of nephrologists reported that they received a higher remuneration fee for managing increasingly complex patients with severe CKD. Conversely, if a physician opts to initiate dialysis on a patient, they are compensated for managing patients thrice weekly as well as being able to more actively monitor patients with potentially high illness acuity. Physician pay-for-performance strategies may be considered but there is limited evidence to suggest that achieving performance measures, such as a target blood pressure goal leads to improvements in patient outcomes in pay-for-performance systems [22], notwithstanding conflicting evidence for focusing on achieving specific targets for surrogate markers in CKD patients. If a patient has a low eGFR (e.g. 8 mL/min/1.73 m^2^) but remains asymptomatic, many nephrologists may wish to follow this patient with regular follow-up visits. A remuneration strategy to compensate nephrologists for this endeavor may be a worthwhile strategy on the part of provincial health authorities given the magnitude of complexity in managing patients with ESRD. As the optimal knowledge translation strategy remains unknown, it remains important that multiple strategies be attempted, each coupled with an appropriate means of evaluation.

Our study had several limitations. First, we administered an online survey which may not capture all of the factors that influence the decision to initiate dialysis in patients with progressive CKD. Our survey pre-dated the release of the Canadian practice guidelines on the initiation of dialysis in patients with progressive CKD. It may be possible that surveyed nephrologists would have answered the questions differently had the guidelines been available to them at the time we disseminated the online survey. The response rate was low and respondents may not be representative of the entire population of Canadian nephrologists; however, there was broad representation from all regions of Canada. In addition, the views of other front-line care-givers, such as advance practice nurses, who may have an impact on the decision to initiate dialysis, were not solicited.

In conclusion, this study demonstrates that the nephrologists consider many clinical and non-clinical factors when deciding on the optimal timing of dialysis initiation, particularly common clinical situations such as uremic symptoms or refractory fluid overload. It also identifies possible areas for improvement. Knowledge translation strategies such as the development and dissemination of guidelines, new assessment tools, implementation and evaluation of a formal review process regarding the timing of dialysis may help frontline nephrologists and patients in the decision-making process of when to initiate dialysis.

## Authors’ information

Interdisciplinary Chronic Disease Collaboration Team: Braden J. Manns, MD, MSc.

## Electronic supplementary material

Additional file 1: **Timing of Dialysis Initiation Survey.** (DOCX 378 KB)
