# Supplementary material for: An assessment of dialysis provider’s attitudes towards timing of dialysis initiation in Canada
Source: Can J Kidney Health Dis. 2014 Apr 7;1:3. doi: 10.1186/2054-3581-1-3 (PMC4346295; doi:10.1186/2054-3581-1-3)
Supplement: Supplementary file 1 — Additional file 1: Timing of Dialysis Initiation Survey. (DOCX 378 KB) [file 40697_2013_3_MOESM1_ESM.docx]

**Supplemental Appendix**

Timing of Dialysis Initiation Survey

We invite you to complete a brief survey seeking information on provider's attitudes towards timing of dialysis initiation. When to start dialysis in patients with progressive chronic kidney disease (CKD) remains controversial as initiation at a higher GFR may be associated with an increase in mortality. The purpose of this survey is to assess national, current practice patterns and the perspectives of dialysis providers regarding the timing of dialysis initiation in Canada. The survey results will be used to determine the need for appropriately tailored knowledge translation activities for the broader nephrology community. All responses will be completely confidential and anonymous. Results will be analyzed in aggregate and at no time will individual responses be made available to anyone other than the investigators.

# Provider Characteristics:

# In what province do you practice?

|  | BC |
| --- | --- |
|  | AB |
|  | SK |
|  | MB |
|  | ON |
|  | QC |
|  | NB |
|  | NS |
|  | NL |
|  | PE |

# In what city do you practice?

# How many years have you been in practice?

|  | 0-5 years |
| --- | --- |
|  | 6-10 years |
|  | 11-15 years |
|  | 16-20 years |
|  | >20 years |

# Do you work primarily in an academic centre or in community practice?

|  | Academic centre |
| --- | --- |
|  | Community practice |
|  | Mixture of both |

# Is your practice located in an urban (population>100,000) or rural (population

|  | Urban |
| --- | --- |
|  | Rural |

# What is the approximate population your hospital/institution provides care for?

|  |  |
| --- | --- |
|  | 50,000-200,000 |
|  | 200,000-500,000 |
|  | >500,000 |

# Do you regularly use a smartphone/PDA device in your practice?

|  | Yes |
| --- | --- |
|  | No |

# Do you use an electronic medical record to assist you in providing care to your CKD patients?

|  | Yes |
| --- | --- |
|  | No |

# In a typical week, how many hours do you devote to journal reading (on-line or in print)?

|  | 0 |
| --- | --- |
|  | 1-2 |
|  | 3-4 |
|  | >4 |

# In a typical month how many hours of CME do you participate in?

|  | 0 |
| --- | --- |
|  | 1-2 |
|  | 2-4 |
|  | >4 |

# In a typical year, how many conferences do you attend?

|  | 0 |
| --- | --- |
|  | 1 |
|  | 2 |
|  | 3 |
|  | >3 |

# Practice characteristics:

# Do you work within a regionally administered renal program (for example province or city-wide) that co-ordinate care of people approaching dialysis?

|  | Yes |
| --- | --- |
|  | No |

# Do you have an education program for patients regarding dialysis +/- modality selection (i.e. group modality education)?

|  | Yes |
| --- | --- |
|  | No |

# Does your centre have a multidisciplinary vascular access clinic?

|  | Yes |
| --- | --- |
|  | No |

# Does your program do preemptive transplantation?

|  | Yes |
| --- | --- |
|  | No |

# Do you have a modality coordinator who sees all (or most patients) and assists with modality selection?

|  | Yes |
| --- | --- |
|  | No |

# Do you have rounds where dialysis modality selection for all new patients are discussed with respect to the optimal dialysis choice?

|  | Yes |
| --- | --- |
|  | No |

# In your province, are physicians reimbursed equally for all dialysis modalities?

|  | Yes |
| --- | --- |
|  | No |

# Is there a higher remuneration fee for care of patients with severe CKD (i.e. eGFR

|  | Yes |
| --- | --- |
|  | No |

# If you answered yes to the question above please describe the differences in fee structure:

# In your centre, approximately how many patients are on:

| CKD stage 5 patients not yet on dialysis? |  |
| --- | --- |
| Peritoneal dialysis? |  |
| Conventional HD? |  |
| At home nocturnal HD? |  |
| In-centre short daily HD? |  |
| Long conventional HD (>5 hours three times a week)? |  |
| Receiving peritoneal and hemodialysis simultaneously? |  |

# Of the available hemodialysis station, what percentage are typically occupied?

|  |  |
| --- | --- |
|  | 51-70% |
|  | 71-90% |
|  | >=91% |

# Timing of dialysis:

# Does your renal program have a formal program-wide policy regarding timing of dialysis initiation?

|  | Yes |
| --- | --- |
|  | No |

# Does your renal program have a review process whereby all new starts are reviewed to assess for timing of dialysis initiation?

|  | Yes |
| --- | --- |
|  | No |

# How do you estimate renal function in late stages of CKD?

|  | eGFR (MDRD/CKD-EPI) |
| --- | --- |
|  | CrCl (C-G formula) |
|  | Serum Cr |
|  | 24 hr urine for CrCl |
|  | 24 hr urine for average of Ucr and Urea |
|  | Plasma urea |
|  | Other |

# Is there an absolute lowest eGFR in an asymptomatic patient on whom you would start dialysis?

|  | 10-12 |
| --- | --- |
|  | 8-10 |
|  | 6-8 |
|  | 4-6 |
|  | 3-4 |
|  |  |
|  | No |

# Does a high number or advanced severity of co-morbidities increase the eGFR you would initiate dialysis in a particular patient?

|  | Yes |
| --- | --- |
|  | No |

# Do uremic symptoms occur earlier in patients with advanced age or more co-morbidity?

|  | Yes |
| --- | --- |
|  | No |

# When would you start a patient on dialysis with a higher eGFR (select all that apply)?

|  | Acute medical condition |
| --- | --- |
|  | Cachexia, not attributed to another cause |
|  | Severe pruritus, not attributed to another cause |
|  | Nausea, not attributed to another cause |
|  | Intended modality |
|  | To avoid emergency |
|  | Patient preference |
|  | Co-morbidity (CHF/CAD/DM/cirrhosis/age) |
|  | Non-compliance |
|  | Fluid overload |
|  | Hyperkalemia refractory to medical therapy |

# Do any of the following influence your recommendation to patients about when to start dialysis (select all that apply)?

|  | Patient preference |
| --- | --- |
|  | Modality |
|  | Social situation |
|  | There is no evidence to support early initiation of dialysis |

# Provider’s attitudes:

# For outpatients with progressive CKD, do you agree or disagree with the following statements?

                                                   Strongly                                                                                                            Strongly                                                    Disagree                                                                                                              Agree

|  | 1 | 2 | 3 | 4 | 5 | 6 | 7 | 8 | 9 |
| --- | --- | --- | --- | --- | --- | --- | --- | --- | --- |
| 1. Starting dialysis with a low eGFR improves patient survival compared to a high eGFR? |  |  |  |  |  |  |  |  |  |
| 2. Starting dialysis with a low eGFR increases hospitalization compared to a high eGFR? |  |  |  |  |  |  |  |  |  |
| 3. Starting dialysis with a low eGFR has a significant negative impact on QOL compared to a high eGFR? |  |  |  |  |  |  |  |  |  |
| 4. Starting dialysis with a low eGFR decreases AVF usage compared to a high eGFR? |  |  |  |  |  |  |  |  |  |
| 5. Starting dialysis with a low eGFR decreases PD utilization compared to a high eGFR? |  |  |  |  |  |  |  |  |  |
| 6. Starting dialysis with a low eGFR leads to sicker patients in general compared to a high eGFR? |  |  |  |  |  |  |  |  |  |
| 7. Starting dialysis with a low eGFR is cost effective compared to a high eGFR? |  |  |  |  |  |  |  |  |  |
| 8. Starting dialysis with a high eGFR is better at preserving RRF compared to a high eGFR? |  |  |  |  |  |  |  |  |  |
| 9. Starting dialysis with a high eGFR is better for PD patients compared to a low eGFR? |  |  |  |  |  |  |  |  |  |
| 10. In terms of clinical outcomes, initiating dialysis at a high eGFR is better than a low eGFR for dialysis initiation? |  |  |  |  |  |  |  |  |  |
| 11. Did the results from the IDEAL trial (showing no difference in mortality between those randomized to a high and low eGFR for initiation of dialysis) make you more likely to delay dialysis initiation in patients with progressive CKD? |  |  |  |  |  |  |  |  |  |
